# Supplementary material for: Revealing the Development Patterns of the Mandibular Glands of Apis mellifera carnica Based on Transcriptomics and Morphology
Source: Insects. 2024 Mar 6;15(3):176. doi: 10.3390/insects15030176 (PMC10971153; doi:10.3390/insects15030176)
Supplement: Supplementary file 1 [file insects-15-00176-s001.zip › insects-2862540-supplementary.pdf]

Table S1. Detailed information on the mapping results of 15 samples.

Sample	Total reads	Map reads	Exon (bp)	Intron (bp)	Intergenic (bp)
AmC_3_1	43,006,096	30,292,759 (70.44%)	4,109,961,386 (90.77%)	125,876,843 (2.78%)	291,950,848 (6.45%)
AmC_3_2	57,254,280	41,508,741 (72.50%)	5,635,682,962 (90.83%)	190,444,901 (3.07%)	378,701,787 (6.10%)
AmC_3_3	58,746,146	44,393,373 (75.57%)	5,993,111,880 (90.33%)	227,085,258 (3.42%)	414,624,196 (6.25%)
AmC_6_1	57,797,970	38,276,176 (66.22%)	5,226,679,862 (91.38%)	168,284,373 (2.94%)	324,892,230 (5.68%)
AmC_6_2	56,128,514	42,254,098 (75.28%)	5,894,096,314 (93.30%)	157,171,821 (2.49%)	265,780,577 (4.21%)
AmC_6_3	61,954,796	48,859,326 (78.86%)	6,776,804,633 (92.78%)	173,597,954 (2.38%)	353,618,795 (4.84%)
AmC_9_1	56,465,200	39,023,097 (69.11%)	5,382,077,998 (92.27%)	175,931,179 (3.02%)	275,028,932 (4.72%)
AmC_9_2	50,420,902	43,842,112 (86.95%)	6,140,046,802 (93.69%)	149,383,738 (2.28%)	264,160,981 (4.03%)
AmC_9_3	49,824,086	39,592,935 (79.47%)	5,474,174,906 (92.58%)	143,704,825 (2.43%)	294,938,278 (4.99%)
AmC_12_1	56,223,708	54,105,789 (96.23%)	7,586,103,383 (93.75%)	197,861,307 (2.45%)	308,095,644 (3.81%)
AmC_12_2	49,313,272	37,084,764 (75.20%)	5,127,975,585 (92.52%)	149,077,241 (2.69%)	265,690,740 (4.79%)
AmC_12_3	62,514,040	49,782,916 (79.63%)	6,828,527,567 (91.84%)	207,485,044 (2.79%)	399,568,071 (5.37%)
AmC_16_1	54,560,816	45,006,942 (82.49%)	6,258,317,884 (93.03%)	152,934,772 (2.27%)	316,069,389 (4.70%)
AmC_16_2	58,984,280	45,740,821 (77.55%)	6,369,915,819 (93.17%)	160,157,760 (2.34%)	307,096,875 (4.49%)
AmC_16_3	50,354,212	43,618,166 (86.62%)	6,069,445,632 (93.09%)	161,276,386 (2.47%)	289,274,156 (4.44%)

Table S2. Information on the primers used in this study.

Gene ID	Sequence (5'to3') F	Sequence (5'to3') R	Production(bp)
GB46290	GCACACTGTAATAGCTTCGAACA	CTTCGAATGCCGGTGTTTCC	118
GB45151	GGAACCGTTTTCGACGAGGA	TGTCCTTGCAGATCTCGCTC	140
GB47301	CCCAACGATCCACCTTACGA	AGTGTCCCGAAAAGAAGCCA	175
GB51787	CGCAGAGGTGGAAGAAGTGT	CTTCTCGAGGTGGAGACTGC	173
GB45400	CTTGCCGGTGATCTGGAAGA	TTCGAAACCATCGTTACATCCA	133
GB42616	CGAGCCTCGACCTTTCAGTT	AAATTTTCGCTATCCGCGCC	112
GB53753	ACAGTTTGCAGGATATGCGGA	GTTCCGGATCGGGTGGTATT	174
LOC102656448	TTGAAACACGCGTGACAATGTA	TCAATGACATCTGGAACAGCA	148
GB47694	GTTGGGGAAGCAATACACGC	ACGGATAAACTCGTCTCGTTC	116
GB47327	ATTTTTACACGTGGACAATGCAG	CAGACACAGTTCGGGTTGTTA	114
GB41227	GGTGTTGTGCCTGCTGATTT	TTTGCAACACTGGCGAACTT	104
GB40240	TCGGACAGTTGGTTCGTTCT	GTGTACTTGGTGCTGGGGTT	177
GB47303	GGGGAAAAACAAAGAGGCGAT	AGCAGTAATTCTTCTTCGATCACT	191
GB42431	TTGGTGGACCAGGATGTGGA	GGACTACCACGGGCAACTTC	115
GB41159	ACATCACAGGAATGCTTTCAAAAT	AAATGGACAACGTTCCCACG	127
